# Feasibility of Adjunctive Bright Light Therapy for Depressive Symptoms on an Acute Psychiatric Floor

**DOI:** 10.7759/cureus.13945

**Published:** 2021-03-17

**Authors:** Alisa Trinh, Pratik Jain, Shaikh Sabahath, Dongliang Wang, James L Megna, Luba Leontieva

**Affiliations:** 1 Psychiatry, State University of New York Upstate Medical University, Syracuse, USA; 2 Public Health and Preventive Medicine, State University of New York Upstate Medical University, Syracuse, USA; 3 Psychiatry and Behavioral Sciences, State University of New York Upstate Medical University, Syracuse, USA; 4 Psychiatry, State University of New York Upstate Medical University, Syracuse, USA

**Keywords:** depression treatment, adjunctive therapy, light therapy, blt, acute psychiatric floor, depression, bright light therapy

## Abstract

Background: Bright light therapy (BLT) has been increasingly used as an experimental treatment in non-seasonal unipolar depression. While clinical trials have demonstrated the efficacy of BLT in ameliorating depression for outpatients, studies examining BLT in the psychiatric inpatient setting are currently lacking.

Aim: The purpose of this study is to explore whether BLT as adjunctive treatment for depressive symptoms on an acute psychiatric floor is feasible and explore associated changes in depressive symptoms.

Methods: An observational, cross-sectional study was conducted at State University of New York (SUNY) Upstate 4B acute inpatient psychiatric unit. BLT was administered to participating patients as adjunctive therapy to their psychopharmacological and psychotherapy treatments on a daily basis throughout their hospitalization. Beck Depression Inventory-II (BDI-II), Hamilton Rating Scale for Depression (HAM-D), and Outcome Questionnaire-45.2 (OQ-45.2) were administered before commencing BLT and after their last BLT session. Changes to the aforementioned measures before and after BLT treatment, the dose response of measure changes based on number of sessions, and the hospital length of stay along with the secondary factors such as age, gender, other psychiatric comorbidities, social factors, and psychiatric medications were analyzed.

Results: BLT is feasible on acute psychiatric inpatient floor with adherence of 94% and has very few side effects. The repeated measures of depression and functioning demonstrated significant decrease in depression and improvement in functioning. Although not statistically significant, clinical meaningful dose-response relationship was found between a number of BLT sessions and improvement in depressive symptoms with five BLT sessions being an optimal amount for depression amelioration.

Conclusion: BLT combined with the ongoing psychopharmacological treatment was well tolerated and easy to administer. It offers a simple, safe, and cost-effective approach to augmenting depressive treatment on an acute psychiatric floor.

## Introduction

Major depressive disorder (MDD) is a common enervating illness affecting 264 million people worldwide [1]. Depression as a chief complaint is prevalent in the inpatient psychiatric unit [2]. Due to significant loss of function and health risks, many people who experience depression need medical treatment. The aim of treatment in the acute phase is to induce remittance of the major depressive episode and attain a return to the patient's baseline functioning [3]. Depressed patients are usually started on antidepressants, such as selective serotonin reuptake inhibitors (SSRIs), which can take a lengthy four-six weeks before antidepressant effects are fully achieved [4].

Many augmentation procedures and non-conventional strategies based on interdisciplinary competencies have been developed to improve the efficacy of antidepressant therapy. A growing body of evidence shows that chronotherapeutic interventions such as bright light therapy (BLT) are useful in treating patients experiencing symptoms of depression [5]. Depression is driven by severe circadian rhythm disruptions for which bright light can be an effective treatment [6].

 BLT was first used in the 1980s as a modality to treat symptoms of depression. Administration of light in the early morning proved to be more effective than increasing amount of exposure time to light in the evening [7]. Currently, light therapy is most known for its use in seasonal affective disorder (SAD) [8], which is a specific type of MDD technically classified as MDD with seasonal pattern under the Diagnostic and Statistical Manual of Mental Disorders, Fifth Edition (DSM-5). SAD is when people experience depression during fall/winter months, which spontaneously remits during spring/summer [9]. Because decreased amount of daylight hours in winter is believed to underlie this seasonal form of depression, several studies have investigated the use of BLT in SAD and have found it to be an effective treatment [8].

A Cochrane review [10] suggested that light therapy is also effective in non-seasonal depression; moreover, its effects are equal to most antidepressant pharmacological therapy trials. Together with standard antidepressant medical treatments, light therapy accelerates recovery, and improvement in depressive symptoms can be noticed within the first week of treatment. After a month of the therapy, patients treated with bright light demonstrated approximately 30% improvement in their depressive symptoms [7].

Despite many lines of evidence indicating the benefits of incorporating BLT in the management of psychiatric disorders, limited data is available as to its use in the inpatient psychiatry unit in the United States. A recent study suggests different factors limit the use of BLT in the inpatient settings such as monetary, regulatory, and organizational restrictions [11]. However, given the negligible non-recurring cost of devices, both hospitals and patients may benefit from the implementation of BLT [11].

A way to augment pharmacotherapy and attain a breakthrough from the debilitating symptoms of depression is needed, especially in the inpatient setting where the most severe cases are treated. Therefore, this study’s main objectives were to investigate the feasibility of implementing BLT as an adjunctive treatment for depression on an acute psychiatric floor and analyze associated changes in depressive symptoms and functioning.

## Materials and methods

Setting

The study took place at a 24-bed acute inpatient psychiatric unit of an academic hospital in a city located in central New York with approximately 147,000 people. The unit admits an average of 75 patients per month. It takes admissions from the emergency room, medical/surgical floors, and the local psychiatric emergency room (at another hospital in the city).

Light intensity was measured at various parts of the psychiatric unit using Extech LT40 LED Light Meter (Extech Instruments, Massachusetts, USA). The corridors had varying light intensities from 50 lux in the corners to 350 lux directly under ceiling lights. Areas receiving the least amount of light were the patient bedrooms receiving 100 lux at bedside level to 120 lux when standing directly under ceiling light. Thus, the unit is considered quite dark, especially in patients’ rooms.

The study began on January 30, 2020 but was temporarily halted on March 16, 2020 due to COVID-related restrictions limiting the number of personnel on-site in the hospital. It was resumed on May 8, 2020 and ended with the last new patient being accepted on August 20, 2020. Over these 150 days in which patients were being enrolled into the study, a total of 326 patients were admitted to the 24-bed acute psychiatric unit and screened for the study through chart review and in-person assessment. This number is comparable to the number of patients admitted in the same time frame (i.e., January 30-March 16 and May 8-August 20) in the previous four years (i.e., 2016-2019), which averages at 328.75 patients.

Design

This was an observational, naturalistic pilot project that used a repeated measures design. There was no random assignment to different groups (i.e., diagnostic groups, medication type groups, etc.). Enrolled participants were followed throughout the course of their treatment as provided by their primary care teams. The project was approved by the State University of New York Institutional Review Board (IRB) and was in accordance with requirements as outlined in the Declaration of Helsinki [12].

Patients already admitted to the acute psychiatric floor were screened for the study through chart review and in-person examinations. Upon their psychiatric hospital admission, eligible patients were approached about the study. Those who consented to the study were given a Beck’s Depression Inventory - Second Edition (BDI-II). Qualifying participants who scored at least a 13 or greater on the BDI-II were enrolled in the BLT study and administered two more depression assessments, which consisted of the Hamilton Depression Rating Scale (HAM-D) and Outcome Questionnaire (OQ-45.2). After completing the three study measures, participants commenced BLT within one to two days of being admitted to the inpatient psychiatric unit. Participants repeated the study measures within 24 hours of hospital discharge. The assessments and screening were conducted by a psychiatry resident and a visiting psychiatrist well-trained in executing all measures.

Bright light therapy sessions

The device used in the study was SunSquare+™ by The SunBox Company (Frederick, Maryland, USA) providing 10,000 lux of light with cost of $360 per lightbox. To ensure participants received the maximum lux, they were positioned approximately 27 inches from the lamp on the high light setting or 17 inches from the lamp on the low light setting. They were permitted to do activities during the sessions if it did not interfere with their eyes' ability to receive the light (e.g., read, work on a puzzle, write, eat, talk, listen to music, etc.).

The BLT sessions took place in a private room with anywhere from one to three participants at a time. The sessions were conducted daily with start times ranging from 7:30 am to 11:30 am. Each session lasted 30 minutes. A study personnel (i.e., psychiatry resident, visiting psychiatrist) monitored participants throughout the BLT sessions for adverse effects and to ensure proper adherence to the BLT protocol. Participants were allowed a maximum of one session per day. They were also allowed to decline or skip any number of sessions.

Measures

1. Beck Depression Inventory - Second Edition (BDI-II): A widely used 21-item self-report inventory to assess the severity of depression with validity and reliability across different populations and cultural groups [13]. Scores range from 0 to 63 where 0-13 indicate minimal depression; 14-19 indicate mild depression; 20-28 indicate moderate depression; and 29-63 indicate severe depression.

2. Hamilton Depression Rating Scale (HAM-D) (17 questions version): A well-researched, clinician-rated scale with established psychometric properties mostly used for depression evaluation [14,15]. Scores range from 0 to 52 where 0-7 indicate no depression; 8-13 indicate mild depression; 14-18 indicate moderate depression; 19-22 indicate severe depression; and >22 indicate very severe depression.

3. Outcome Questionnaire (OQ-45.2): Assesses the level of symptom distress, interpersonal functioning, and social role [16]. Total scores range from 0 to 180 where >63 indicate clinical concern; symptom distress scores range from 0-100 where >36 indicate clinical concern; interpersonal relations scores range from 0 to 44 where >15 indicate clinical concern; and social role scores range from 0 to 36 where >12 indicate clinical concern.

Statistical analysis

For descriptive purposes, categorical variables are summarized by frequencies (proportions), and continuous variables are summarized by mean (standard deviation) and median (range). To test the difference of categorical variables between groups, Pearson chi-square tests are primarily used. Still, Fisher exact tests may be used instead if more than 50% of the contingency tables' cell counts have expected cell counts less than 5. Two-sample t-tests are used to compare the difference of continuous variables. Pearson correlation coefficient was used to assess the relationship between number of BLT treatment and changes in outcome measures. One-way analysis of variance (ANOVA) was used to compare more than two groups. All analyses are performed using SAS 9.4 (SAS Institute, Cary, North Carolina, USA). Wilcoxon rank sum tests are used as an alternative to t-test if the sample sizes of at least one group are below 20.

## Results

**Figure 1:**
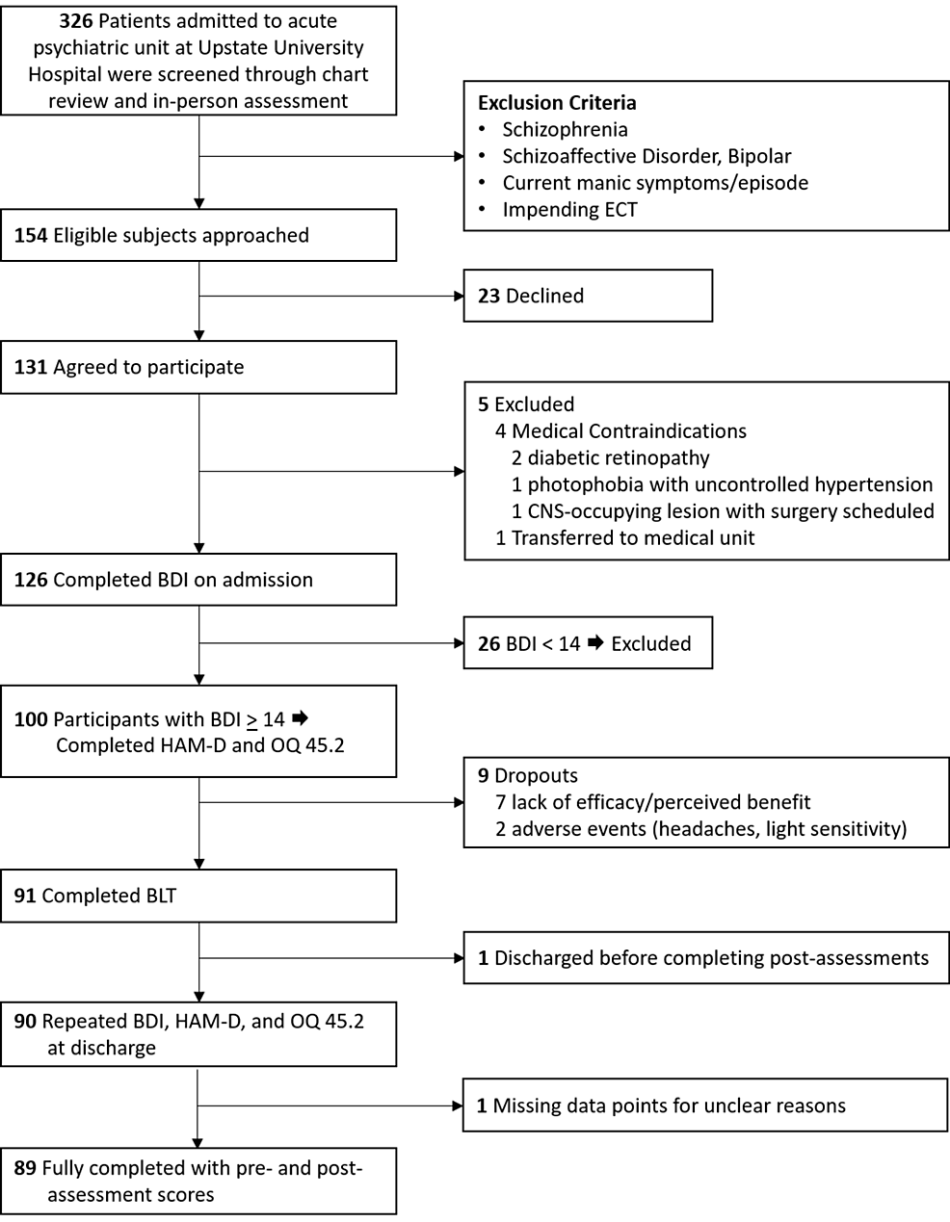
Study flow ECT, Electroconvulsive therapy; BLT, bright light therapy; BDI, Beck Depression Inventory; HAM-D, Hamilton Depression Rating Scale; OQ 45.2, Outcome Questionnaire 45.2.

Study flow of patients and inclusion/exclusion criteria are presented in Figure 1. Out of the 326 patients screened, 47% (154) were found to be eligible and offered the option to participate in the study, and 85% (131) patients consented to the study, while 15% (23) patients declined. From the 131 participants, four were excluded due to medical contraindications (see Figure 1), one was transferred to a different unit, and 26 did not score high enough on the BDI-II to qualify for the study. Thus, out of 154 potentially eligible patients, 65% (100) patients qualified with a BDI-II score > 13 and completed two additional assessments (HAM-D and OQ 45.2) before starting their daily BLT. Out of the 100 subjects, 9% dropped out of BLT. Seven patients dropped out due to lack of perceived benefit, one due to headache, and one due to bright light sensitivity. There were no serious adverse events during this project. Out of the 91 subjects who completed BLT, there was a session adherence rate of 94%.

Demographic characteristics of eligible subjects are categorized in Table 1. There were slightly more females, singles, and unemployed persons among those who completed the BLT study. The majority were Caucasians, domiciled, and completed at least 10-12 years of education. The mean age of participants was mid-thirties.

**Table 1:** Demographic characteristics of eligible subject ISCED, International Standard Classification of Education.

Characteristic	Categories	Completed (N = 90)	Declined or Dropped Out (N = 31)
Age	Mean (SD)	34.5 (14.4)	42.3 (16.3)
	Median (Range)	32 (18-70)	40 (18-71)
Sex	Male	36 (40%)	18 (56%)
	Female	53 (59%)	13 (41%)
	Trans	1 (1%)	1 (3%)
Race	Caucasian/White	76 (84%)	27 (90%)
	African-American/Black	11 (12%)	1 (3%)
	Hispanic	2 (2%)	2 (7%)
	Native American	1 (1%)	0 (0%)
Marital Status	Single	46 (51%)	16 (57%)
	Married	36 (40%)	9 (32%)
	Divorced	8 (9%)	3 (11%)
Housing	Domiciled	83 (92%)	27 (90%)
	Homeless/Shelter	7 (8%)	3 (10%)
Employment	Employed	24 (27%)	10 (33%)
	Unemployed/Disability Benefits	53 (59%)	15 (50%)
	Student	8 (9%)	4 (13%)
	Retired	5 (6%)	1 (3%)
Education Level (ISCED)	2 (7^th^-9^th^ grade)	5 (6%)	6 (21%)
	3 (10^th^-12^th^ grade)	56 (62%)	12 (43%)
	4 (Associate’s Degree, 2-year college)	18 (20%)	3 (11%)
	5 (Bachelor’s Degree, 4-year college)	10 (11%)	5 (18%)
	6 (Master’s or Doctorate’s Degree)	1 (1%)	2 (7%)

The most common psychiatric diagnoses of subjects in our study are displayed in Table 2. Many patients had more than one psychiatric diagnosis. Almost half of the participants had MDD and/or an active substance use disorder, one-third had post-traumatic stress disorder, and one-third had borderline personality disorder.

**Table 2:** Psychiatric diagnoses of subjects *Other diagnoses include adjustment disorder, generalized anxiety disorder, persistent depressive disorder, and bipolar disorder.

Diagnosis	Completed	Declined or Dropped Out
Major Depressive Disorder	36 (40%)	12 (40%)
Post-Traumatic Stress Disorder	26 (29%)	2 (7%)
Borderline Personality Disorder	24 (27%)	6 (20%)
Active Substance Use Disorders	40 (44%)	17 (57%)
Other*	21 (23%)	4 (13%)

Comorbid substance abuse or use disorders are listed in Table 3. Table 2 includes only patients with substance use disorders that are active, whereas Table 3 includes patients with the specified substance use disorder that are either active or in remission per DSM-5 criteria. It was not uncommon for patients to abuse more than one substance. Cannabis was the most common substance abused followed by nicotine and alcohol among participants.

**Table 3:** Substance abuse (active or in remission) of subjects

Substance	Completed	Declined or Dropped Out
Alcohol	30 (33%)	17 (55%)
Tobacco/Nicotine	40 (44%)	18 (58%)
Cannabis	47 (52%)	14 (45%)
Opioids	12 (13%)	4 (13%)
Hallucinogens	14 (16%)	0 (0%)
Cocaine	16 (18%)	9 (29%)
Other Stimulants (e.g., methamphetamine)	10 (11%)	2 (6%)

Scheduled psychiatric medications of the subjects at time of discharge are displayed in Table 4. The most common medications were SSRIs, followed by atypical antipsychotics, and trazodone.

**Table 4:** Psychiatric medications of subjects

Medication Class	Completed	Declined or Dropped Out
Selective Serotonin Reuptake Inhibitors	52 (58%)	14 (50%)
Serotonin Norepinephrine Reuptake Inhibitors	10 (11%)	3 (11%)
Norepinephrine Dopamine Reuptake Inhibitors	1 (1%)	1 (4%)
Tricyclic Antidepressants	5 (6%)	0 (0%)
Trazodone	22 (24%)	2 (7%)
Mirtazapine	8 (9%)	2 (7%)
Benzodiazepines	4 (4%)	1 (3%)
Other Sedative/Hypnotics	20 (22%)	6 (21%)
Typical Antipsychotics	3 (3%)	2 (7%)
Atypical Antipsychotics	30 (33%)	8 (29%)
Mood Stabilizers	15 (17%)	6 (21%)
Naltrexone	15 (17%)	5 (17%)

Participants received an average of 5.51 BLT sessions (SD = 4.09) with an average length of stay in the hospital of 6.47 days (SD = 4.93). See Table 5. One outlier was removed, which was a patient with an extensive length of stay awaiting state hospital transfer.

**Table 5:** Clinical characteristics of participants *Missing number of BLT sessions for a participant who completed BLT. BLT, Bright light therapy.

	Completed	Completed Excluding Outlier	Dropped Out
Length of Stay (Days in the Hospital)	N = 91	N = 90	N = 9
Mean (SD)	7.26 (8.90)	6.47 (4.93)	10.0 (7.43)
Median (Range)	6 (1-77)	6 (1-32)	7 (1-23)
Number of BLT Sessions	N = 90*	N = 89*	N = 9
Mean (SD)	6.17 (7.47)	5.51 (4.09)	2.0 (1.6)
Median (Range)	5 (1-65)	5 (1-25)	1 (1-5)

Participants tolerated BLT well. There were five patients who complained of headache and three of eye strain, which disappeared with the decreased light intensity from 10,000 to 5,000 lux. Most of these patients were able to later tolerate the light at 10,000 lux without any issues. There were nine patients who dropped out of BLT - all of whom cited lack of perceived benefit with the exception of one patient who dropped out due to adverse effects of headache and eye strain. No significant side effects were reported or observed throughout the study.

Table 6 details the changes in study measures for BLT participants. All study measures demonstrated statistically significant improvement over the course of BLT. Self-reported depressive symptoms (BDI-II) and HAM-D scores decreased by over half. Patients also indicated significantly improved symptoms distress, interpersonal relationships, and social functioning.

**Table 6:** Assessment score changes during hospitalization

	Baseline	Discharge	Mean Difference (95% CI)	Percentage Change	p Value
Beck Depression Inventory			-15.5 (-18.0, -12.8)	-59.5%	<0.0001
N	89	89			
Mean (SD)	31.3 (10.60)	15.8 (11.95)			
Median (Range)	32 (14-63)	13 (0-49)			
Hamilton Depression Scale			-13.4 (-14.8, -12.0)	-60.2%	<0.0001
N	89	89			
Mean (SD)	22.1 (5.36)	8.8 (6.32)			
Median (Range)	23 (8-35)	8 (0-27)			
Outcome Questionnaire 45.2					
Total Score			-26.8 (-31.8, -21.7)	-27.9%	<0.0001
N	89	89			
Mean (SD)	95.8 (24.92)	69.1 (28.56)			
Median (Range)	97 (28-165)	69 (12-138)			
Symptoms Distress			-18.1 (-21.3, -14.9)	-30.4%	<0.0001
N	89	89			
Mean (SD)	59.5 (14.87)	41.4 (17.45)			
Median (Range)	60 (15-94)	41 (6-77)			
Interpersonal Relationship			-4.3 (-5.7, -2.9)	-21.3%	<0.0001
N	89	89			
Mean (SD)	20.7 (8.09)	16.3 (7.94)			
Median (Range)	21 (2-43)	16 (1-37)			
Social Role			-3.8 (-5.2, -2.5)	-24.2%	<0.0001
N	89	89			
Mean (SD)	15.7 (6.75)	11.9 (6.29)			
Median (Range)	15 (2-32)	12 (0-28)			

A greater number of BLT sessions was correlated with a more significant decrease or improvement in depression and functioning assessment scores, with BDI-II and HAM-D reaching near statistical significance, as shown in Table 7.

**Table 7:** Correlation between number of BLT sessions and change in assessment scores (post-pre) BDI-II, Beck Depression Inventory-II; HAM-D, Hamilton Depression Rating Scale; OQ-45.2, Outcome Questionnaire-45.2, BLT, bright light therapy.

Assessment	Pearson Correlation Coefficient	p Value
BDI-II	-0.14	0.073
HAM-D	-0.15	0.058
OQ-45.2 Total	-0.12	0.129

**Figure 2:**
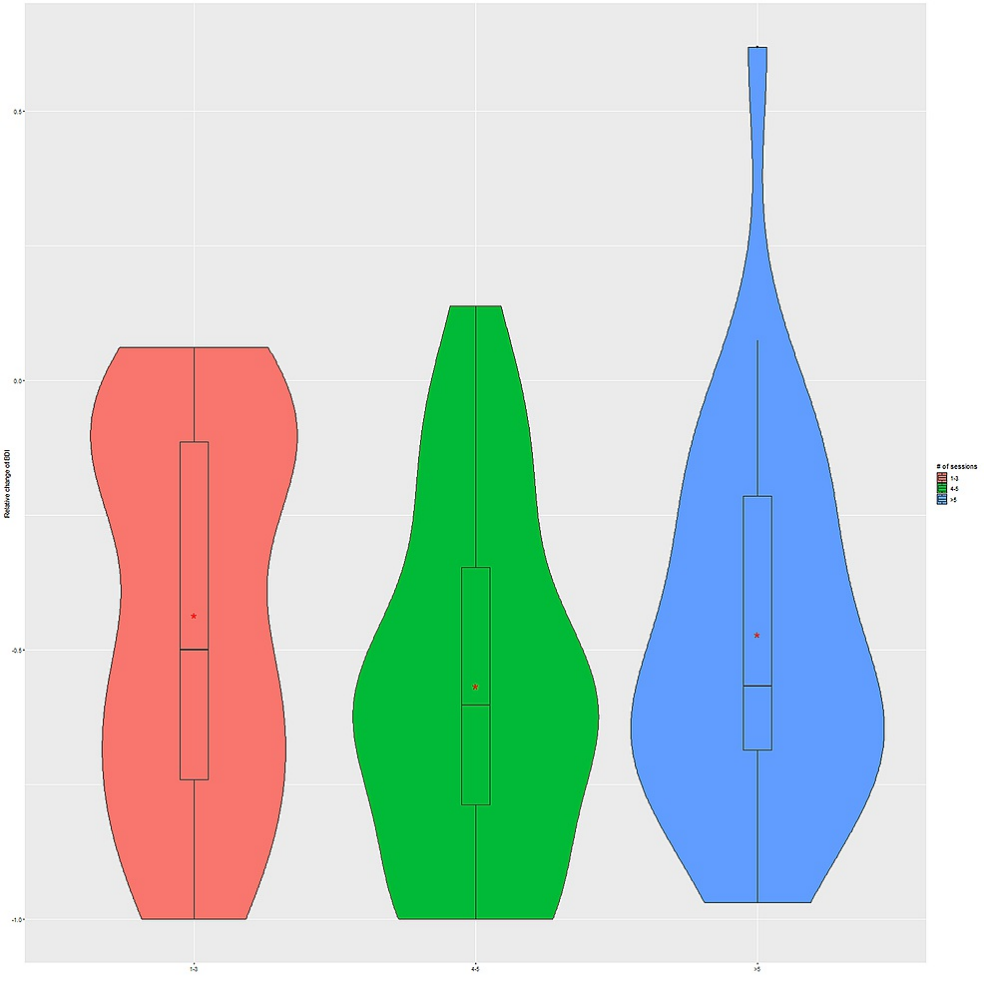
Dose-response relationship between the number of BLT sessions and BDI-II score change The (*) indicates average values, and the first, second, and third quartiles are shown in the boxes. BLT, Bright light therapy; BDI-II, Beck Depression Inventory-II.

**Figure 3:**
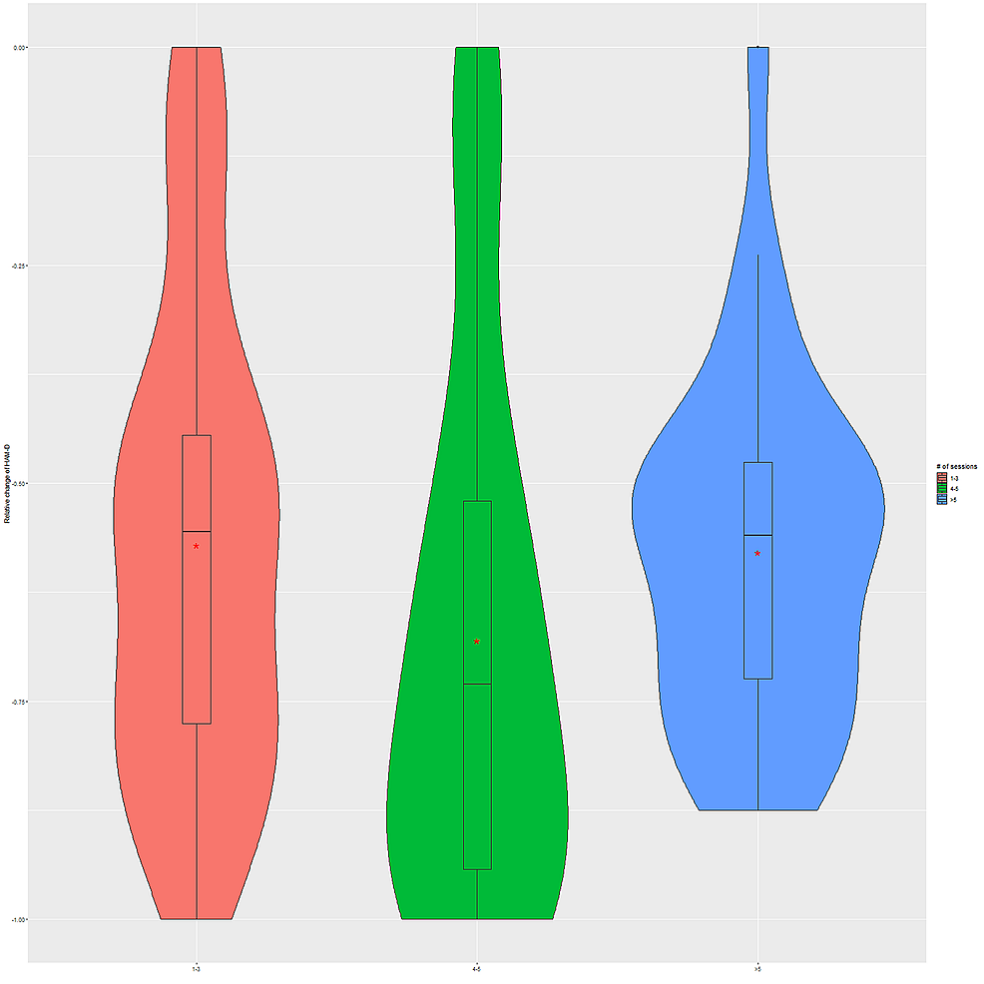
Dose-response relationship between the number of BLT sessions and HAM-D score change The (*) indicates average values, and the first, second, and third quartiles are shown in the boxes. BLT, Bright light therapy; HAM-D, Hamilton Depression Rating Scale.

More in-depth analysis of the relationships between the number of BLT sessions and relative changes in BDI-II and HAM-D measures suggests that four to five sessions were the most beneficial for the improvement in depression scores (see Figures 2, 3, respectively). The relative change was defined as (post-pre)/pre. The findings do not reach statistical significance for both BDI-II (F = 1.26, p = 0.290) and HAM-D (F = 1.6, p = 0.208) from one-way ANOVA analysis.

## Discussion

While there have been clinical trials to demonstrate the efficacy of BLT in the outpatient setting [17,18], this observational feasibility study represents a rare investigation to directly examine BLT on the acute inpatient psychiatric floor. Sunlight may have important implications in the recovery of patients in the hospital as suggested in a recent study, which found that patients admitted in rooms with more sunlight exposure were discharged earlier than patient in rooms with less sunlight exposure [19]. Of note, the psychiatric unit at SUNY Upstate Hospital is dark compared to standards [20] and receives no direct sunlight during winter months. Additionally, admitted psychiatric patients at this hospital are restricted to the unit, and outdoor access is prohibited throughout their hospitalization for several safety reasons. Patients on the psychiatric unit at Upstate spend majority of their time in their rooms where lighting is most minimal with an average of 100 lux in all rooms at bedside. Hence, such patients have significantly lower light exposure of 50-500 lux compared to the average outdoor sunlight exposure, which ranges from 1,000-100,000 lux. Other psychiatric facilities in urban settings face similar issues where patients are markedly deprived of sunlight by surrounding buildings and outdoor access restrictions. This void of natural light could be filled by BLT.

Overall, our data indicate that BLT was well-received by patients: 85% of eligible patients approached consented. Only 9% of subjects dropped out of BLT, whereas 91% continued BLT throughout their hospitalization. Among those that completed BLT, there was a 94% BLT session adherence rate. During the BLT study, participants self-reported significant functional improvement with a decline in depressive symptoms as demonstrated by the decrease in BDI-II, OQ-45.2, and HAM-D scores from pre- to post-BLT time points. Although there was no control group, previous studies conducted in the outpatient setting have documented substantial, more rapid clinical benefit, with consistent effects using BLT as an augmentation to pharmacotherapy [17,18]. A clinically meaningful, although not statistically significant, dose-response relationship was observed with an average of five BLT sessions being the most beneficial in alleviating depressive symptoms. This further underscores the applicability of BLT for the psychiatric unit given its mean length of stay of 7.4 days with a median of five days [3].

In our study, BLT has proven to be largely safe with minimal side effects. Previous studies have noted no significant differences between side effects of placebo versus BLT [21]. This is consistent with our study findings of only minor reported side effects: headache and eye strain among five and three participants, respectively. These side effects disappeared upon decreasing light intensity from 10,000 lux to 5,000 lux. Majority of these same participants were able to later tolerate the light at 10,000 lux without any issues. Only one patient dropped out due to the adverse effects (eye strain and headache), whereas the rest of the 9% (eight patients) who dropped out of the BLT study merely cited lack of perceived benefit.

In addition to generating few adverse effects, BLT did not interfere with any other psychiatric treatments. Another advantage of BLT is the fairly inexpensive cost. The price for one SunBox lamp is $475 with lightbulbs guaranteed to last for at least two years, which can easily serve hundreds of patients over the course of its lifetime [22].

In terms of limitations, one the greatest limiting factor for this BLT feasibility study was staff allocation. Because the lightbox has an electrical cord that poses as a potential tool for strangulation, regulations on the psychiatric unit dictate that patients must be supervised throughout the BLT session. In our study, we found that nurses on the floor were too busy to adequately monitor patients during their BLT sessions. Thus, the addition of a staff member dedicated to monitoring patients during BLT sessions was required (which in the case of our study was simply the psychiatry resident conducting the research). However, technological advancements now make it possible to control artificial lighting remotely. Intensity-controlled lighting can be installed, especially by the bedside, further increasing the feasibility of implementing BLT on the acute psychiatric floor.

## Conclusions

BLT is a non-invasive adjunctive therapy for psychotherapy and pharmacotherapy treatment targeting depressive symptoms that was well-received by a large majority of the patients in our study. It is highly feasible on an acute psychiatric unit, given the lightbox’s inexpensive costs, ready availability, and minimal side effect profile. However, our study had a small sample size, limited duration, and lack of control group. Given its feasibility and potential to ameliorate depressive symptoms, further research investigating the efficacy of adjunctive BLT in the treatment of depression in the acute psychiatric inpatient setting should be implemented.
